# Frequent mutations in acetylation and ubiquitination sites suggest novel driver mechanisms of cancer

**DOI:** 10.1186/s13073-016-0311-2

**Published:** 2016-05-12

**Authors:** Soumil Narayan, Gary D. Bader, Jüri Reimand

**Affiliations:** The Donnelly Centre, University of Toronto, Toronto, Canada; Present address: Ontario Institute for Cancer Research, Toronto, Ontario Canada; Present address: Department of Medical Biophysics, University of Toronto, Toronto, Ontario Canada

## Abstract

**Background:**

Discovery of cancer drivers is a major goal of cancer research. Driver genes and pathways are often predicted using mutation frequency, assuming that statistically significant recurrence of specific somatic mutations across independent samples indicates their importance in cancer. However, many mutations, including known cancer drivers, are not observed at high frequency. Fortunately, abundant information is available about functional “active sites” in proteins that can be integrated with mutations to predict cancer driver genes, even based on low frequency mutations. Further, considering active site information predicts detailed biochemical mechanisms impacted by the mutations. Post-translational modifications (PTMs) are active sites that are regulatory switches in proteins and pathways. We analyzed acetylation and ubiquitination, two important PTM types often involved in chromatin organization and protein degradation, to find proteins that are significantly affected by tumor somatic mutations.

**Methods:**

We performed computational analyses of acetylation and ubiquitination sites in a pan-cancer dataset of 3200 tumor samples from The Cancer Genome Atlas (TCGA). These analyses were targeted at different levels of biological organization including individual genes, pathway annotated gene sets, and protein-protein interaction networks.

**Results:**

Acetylation and ubiquitination site mutations are enriched in cancer with significantly stronger evolutionary conservation and accumulation in protein domains. Gene-focused analysis with the ActiveDriver method reveals significant co-occurrences of acetylation and ubiquitination PTMs and mutation hotspots in known oncoproteins (TP53, AKT1, IDH1) and highlights candidate cancer driver genes with PTM-related mechanisms (e.g. several histone proteins and the splicing factor SF3B1). Pathway analysis shows that PTM mutations in acetylation and ubiquitination sites accumulate in cancer-related processes such as cell cycle, apoptosis, chromatin regulation, and metabolism. Integrated mutation analysis of clinical information and protein interaction networks suggests that many PTM-specific mutations associate with decreased patient survival.

**Conclusions:**

Mutation analysis of acetylation and ubiquitination PTM sites reveals their importance in cancer. As PTM networks are increasingly mapped and related enzymes are often druggable, deeper investigation of specific associated mutations may lead to the discovery of treatment-relevant cellular mechanisms.

**Electronic supplementary material:**

The online version of this article (doi:10.1186/s13073-016-0311-2) contains supplementary material, which is available to authorized users.

## Background

Cancer is a complex, heterogeneous class of diseases with the highest mortality in the developed world. Cancer is driven by molecular alterations that activate oncogenes and inhibit tumor suppressor genes. These cancer driver events modify cellular pathways and provide cells with selective advantages such as uncontrolled growth, suppressed apoptosis, replicative immortality, and mobility [1]. Different classes of driver modifications are recognized, including chromosomal copy number alterations [2], epigenetic modifications in DNA methylation [3] and chromatin state [4], and DNA mutations such as short insertions-deletions and single nucleotide variants (SNVs). SNVs are abundant mutations that are reliably detected in paired sequencing of germline and tumor DNA. The Cancer Genome Atlas (TCGA) and the International Cancer Genome Consortium (ICGC) compile comprehensive molecular profiles of cancer genomes, transcriptomes, and epigenomes for many tumor types [5, 6]. Integrative analysis of these profiles can provide insights into tumor biology and support the development of cancer therapies.

Discovering drivers of tumor-specific phenotypes is an important goal of cancer genomics [6]. Driver genes are challenging to find, as tumor genomes also contain abundant functionally neutral passenger mutations acquired as a result of genome instability. It is estimated that a typical solid tumor carries 2–8 driver mutations and ten times more passengers [7]. Analysis of positive selection assumes that frequent recurrence of a particular mutation in independent patient samples is statistically unlikely and therefore indicates a cancer driver. Tools such as MutSig and MuSic predict genes where observed mutations significantly exceed expected genome-wide mutation rates [8, 9]. Such approaches are complicated by mutational heterogeneity, as mutation rates vary across genes, and nucleotide signatures [8, 10, 11], and are influenced by factors such as gene expression intensity [12], DNA replication timing [13], and chromatin organization [14]. Further, frequency-based methods do not provide corresponding mechanistic hypotheses to explain the functional consequence of a mutation.

We hypothesize that many functional cancer mutations alter protein-protein interaction networks and associated biochemical mechanisms [15]. Post-translational modifications (PTMs) are chemical alterations of amino acids (AAs) that act as regulatory switches, extending the functional repertoire of proteins and regulating protein interactions in cell-signaling networks [16]. Thus, specific mutations in PTM sites may alter networks and lead to changes in cellular phenotype involved in disease development. We recently developed ActiveDriver, a site-specific mutation enrichment model that highlights cancer genes with significant co-occurrence of missense mutations and PTM sites [17]. ActiveDriver assumes that many independent mutations in patient tumors are unlikely to co-occur in PTM sites of the protein unless these sites are important for the protein’s function in cancer. In estimating mutation significance, ActiveDriver uses a Poisson regression model that accounts for multiple factors including protein disorder, direct and flanking PTM residues, and site density. ActiveDriver compares substitution rates in individual proteins with substitutions in their active sites and thus provides complementary information to traditional genome-wide mutation models. Our previous analyses of phosphorylation-related mutations predicted novel cancer genes and pathways, mechanistic hypotheses of mutation function, and found significant clinical correlations [17–19]. Site-specific mutation analysis is thus a viable strategy to discover putative cancer driver genes and hypothesize how they biochemically function to drive cancer.

Here we focus on acetylation and ubiquitination, two PTMs of lysine (K) residues. These are the best-characterized PTMs after phosphorylation and abundant experimental data are available for human proteins [20–22]. Acetylation and ubiquitination involve distinct electro-chemical mechanisms and biological roles, and their co-occurrence on lysines leads to cross-talk and combinatorial switches of PTMs [23]. Moreover, PTM sites have a tendency to cluster in protein sequence, and mutations in regions with highly overlapping PTM sites create the potential for mutations to target multiple sites [24]. Here we refer to ubiquitination and acetylation as lysine PTMs or PTMs unless otherwise indicated.

Acetylation is the reversible addition of acetyl groups to lysines conducted by two families of enzymes, acetyltransferases (HATs), and deacetylases (HDACs). Acetylation is primarily associated with chromatin regulation. The histone code of PTMs determines DNA packaging, resulting in open or closed chromatin conformations and modulation of gene expression [25]. Specific histone modifications and cancer mutations are associated with silencing of tumor suppressor genes and cancer progression [26, 27]. Growing evidence indicates that non-histone proteins, in particular transcription factors, are subject to acetylation [28]. Acetylation is increasingly clinically targetable, as pharmaceutical inhibition of HDAC enzymes for cancer therapy has been subject to recent clinical trials [29].

Ubiquitination is the addition of ubiquitin peptides to lysines coordinated by ubiquitin ligases and related enzymes. This PTM is canonically the signal of proteasomal degradation, although other roles are known [30, 31]. Ubiquitination is implicated in many processes, including receptor endocytosis, proteolysis, protein trafficking, inflammation, translation, and DNA repair. Ubiquitination also occurs in histones and interacts with other PTMs of the histone code [25]. It is implicated in tumor development and the ubiquitin pathway is an emerging drug target [32].

As lysine PTMs are involved in cancer-related processes, we hypothesize that corresponding signaling networks are altered in cancer cells by specific mutations in enzymes and substrate sites. Two recent studies surveyed the pan-cancer mutational landscape of epigenetic regulators [33, 34]; however, no comprehensive analysis of cancer mutations affecting acetylation and ubiquitination sites is available. Thus, we collected cancer mutations in acetylation and ubiquitination sites and examined their proteome-wide properties and occurrence in cancer driver genes, pathways, and molecular interaction networks. As these PTMs are prospective targets in drug development, this information helps discover cancer driver mechanisms with testable hypotheses of pharmacological modulation.

## Methods

### Post-translational modifications and cancer mutations

Acetylation and ubiquitination sites for human proteins were retrieved from the PhosphoSitePlus database (retrieved 2013-05-23) [22] and filtered to only keep data from peer-reviewed publications. PTM sites defined as 15-mer AA sequences were mapped exactly to 18,671 high confidence sequences of longest protein isoforms of the Consensus Coding Sequence (CCDS) database. Sites with multiple matches per protein were considered, while partially mapping sites were discarded. Shared PTM sites comprised sites where central lysines associated with both acetylation and ubiquitination.

We retrieved the pre-processed pan-cancer dataset of somatic mutations in 3185 tumor samples curated by the TCGA for 12 different cancer types [5, 35] from the Synapse repository (ID syn1729383). We removed 91 hyper-mutated samples with an extreme number of mutations. Chromosomal coordinates of mutations were converted to protein-level substitutions using the Annovar software [36] and filtered for the longest isoforms defined above. We only considered non-synonymous missense SNVs and discarded all other types including nonsense, silent, insertion/deletion, and non-coding mutations.

### Selection, conservation, and domain associations of PTM site mutations

Positive selection of PTM sites in cancer genomes was evaluated separately for acetylation and ubiquitination sites. We compared observed and expected numbers of mutations in PTM-associated and non-associated protein sequences and computed significance with one-sided bootstrap tests with 100,000 shuffles per test. For both types of PTMs, we only focused on proteins with these modifications and discarded other non-modified proteins.

Protein domain information for the CCDS sequences was retrieved from the databases Pfam [37] and SMART [38]. Disordered sequence regions of proteins were predicted with the DISOPRED2 software [39] using default parameters (Additional file 1). Domain-associated and non-domain sequence regions were assessed for PTM-related and non-related mutations, and expected distributions of mutation rates were derived by resampling of sequence residues. Mutation rates of domain and non-domain regions were tested with the non-parametric one-sided bootstrap tests with 100,000 shuffles per test.

Protein sequence conservation corresponds to phastCons 46-way scores [40] derived from the Annovar software. We compared conservation scores of mutated AAs in PTM-related and non-PTM sequence separately for disordered and structured protein sequences with the non-parametric one-sided bootstrap test with 100,000 shuffles per test.

### ActiveDriver analysis of PTM mutations and recurrently mutated sites

Cancer mutations were mapped to PTM sites and PTM-specific significance scores of genes were computed with the ActiveDriver software [17]. Mutations were classified by their proximity to central lysine residues, into three categories: (1) direct mutations on the central lysine; (2) proximal mutations within 1–2 residues of the central lysine; and (3) distal mutations within 3–7 residues of the central lysine, as defined by the default settings of ActiveDriver. Mutational significance was estimated across all cancer samples of 12 types. Genes with false discovery rate (FDR) *p* <0.05 were considered significant. We used the OncoDriveClust method [41] to assess clustering of mutations in all genes including results from ActiveDriver, using the recommended settings for TCGA data from OncoDriveClust documentation. To estimate the significance of known cancer genes in ActiveDriver results, we implemented a custom permutation test with mutation frequency of genes. In brief, we binned all genes into 100 groups according to mutation frequency and then sampled the number of genes detected by ActiveDriver, according to the expected mutation frequency distribution of known cancer genes. We estimated an empirical *p* value from 10 million permutations relative to the number of cancer genes detected in ActiveDriver results according to the Cancer Gene Census database [42].

### Pathway analysis of PTM mutations and Enrichment Map

Pathway analysis was performed using functional annotations from the Gene Ontology [43], and the databases of Reactome [44] and CORUM [45], retrieved from the g:Profiler webserver [46]. The protein sets were filtered to discard small (≤2 proteins) and large lists (>1000 proteins). Only annotations of biological processes of the Gene Ontology were used. Pathway analysis considered proteins with PTM sites while non-modified proteins were discarded. Each gene list corresponding to a pathway or process was tested separately with a Poisson exact test. Observed protein sequence length and associated mutations of PTM sites of protein sets were compared with the expected mutation rate of all pathway-associated protein sequence. Protein sets with fewer than two separately mutated proteins were discarded to avoid results from single cancer drivers. Pathways with an FDR-corrected *p* value <0.05 were considered significant. Pathways were visualized using the Enrichment Map app [47] of the Cytoscape software [48] that visualizes enrichment analysis results as a network with nodes representing pathways and edges connecting pathways with many shared genes. Singleton nodes and small and redundant groups of up to eight nodes were discarded for simplicity and the remaining sub-networks were manually curated and assigned the most representative functional annotations.

### HyperModules analysis of protein networks and patient survival information

To analyze patient survival information associated with PTM mutations, we constructed a PTM-specific interaction network of human proteins from the BioGRID database [49]. We selected physical protein-protein interactions between proteins with PTM sites and proteins annotated as PTM enzymes. Interactions for acetylation and ubiquitination were compiled separately. PTM enzymes were curated from three resources: the Gene Ontology [43], the hUbiquitome database [50], and the Compendium of Protein Lysine Acetylation (CPLA) [20]. Survival information of patients profiled by the TCGA were retrieved from the study by Kandoth et al. [51]. Patients with each type of cancer were analyzed separately. Survival-associated network modules were predicted with the Cytoscape implementation of the HyperModules algorithm [52]. HyperModules is a greedy method to find maximally survival-correlated modules that uses a log-rank test to evaluate significance of survival correlations. The modules were additionally filtered after expected distributions of *p* values were computed from 100 network-shuffling permutations. Modules with empirical *p* <0.05 from permutation tests were considered significant.

## Results

### Acetylation and ubiquitination sites are altered by thousands of cancer mutations

To characterize cancer mutations in acetylation and ubiquitination sites, we collected experimentally determined sites in human proteins from the PhosphoSitePlus database [22]. We retrieved 29,933 sites in 7167 proteins, including 7480 acetylation sites, 25,773 ubiquitination sites, and 3280 sites targeted by both PTMs. We included fourteen (± seven) residues of flanking sequence around PTM sites and merged regions of overlapping PTM sequence. Flanking sequence affects interactions of substrates and PTM enzymes in phosphorylation [53] and acetylation [54]; however, less is known about the specificity of ubiquitination enzymes. Flanking sequence also associates with short linear motifs and PTM cross-talk, potentially altered by mutations in cancer.

We integrated PTM sites with cancer mutations, using the dataset of 241,701 somatic missense SNVs in 3185 tumor samples of the TCGA pan-cancer project comprising 12 cancer types including brain, colon, and lung cancer [5]. For each tumor sample the TCGA provided a normal blood (or tissue) sample to serve as a control [5]. We found 2106 acetylation-related SNVs, 6405 ubiquitination-related SNVs, and 883 SNVs in shared sites of the two PTMs, covering 72 % of cancer samples (Additional files 2 and 3). When considering only lysine residues, there are a total of 8381 SNVs of which 599 are associated with PTM sites. PTM mutations replacing the central lysine residues are most likely to disrupt PTMs and we refer to these as direct mutations. The majority of mutations occur in flanking sequence of PTMs and potentially affect protein modification indirectly through enzyme sequence specificity and local structural environment.

### Lysine PTM sites are enriched in cancer mutations with high conservation and functional impact

**Fig. 1:**
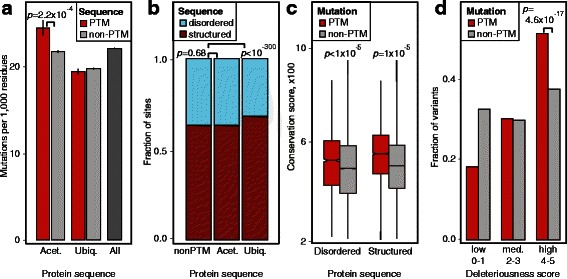
Importance of cancer mutations in post-translational modification (PTM) sites of acetylation and ubiquitination. **a** Cancer mutations in protein acetylation sites are significantly more frequent than non-modified protein sequence, while ubiquitination sites show expected mutation rates. PTM sites include central lysine residues and ±7 flanking windows. Comparisons only include proteins with respective PTM sites. **b** Ubiquitination sites are enriched in protein sequences associated with structured regions, while acetylation sites are evenly distributed among structured and disordered regions. **c** PTM-associated cancer mutations show greater evolutionary conservation than non-PTM mutations. Disordered and structured protein sequences are compared separately. **d** PTM-associated cancer mutations are more frequently predicted deleterious by an ensemble of five variant function predictors

We studied proteome-wide properties of PTM-related cancer mutations to evaluate their global functional importance in cancer. We focused on proteins with at least one PTM site and removed non-modified proteins to avoid systematic biases. First, we examined substitution rates in ubiquitination and acetylation sites (including their ±7 flanking region) in comparison to non-PTM sequence. We found that acetylation sites are enriched in cancer mutations (fold change [FC] = 1.12, *p* = 2.2 × 10^–4^, bootstrap test) while ubiquitination PTM sites show no significant difference in mutational frequency (FC = 0.98, *p* = 0.12, Fig. 1a). In addition, we studied rates of substitution for the central modified lysine residues with non-modified lysines, and found that substitutions of central PTM residues are more frequent than expected (FC = 1.21, *p =* 2.9 × 10^–2^ for acetylation; FC = 1.13, *p* = 6.0 × 10^–3^ for ubiquitination). This suggests that rewiring of lysine PTMs is important in cancer and this is not an artefact of codon structure. Interestingly, proteins with lysine PTMs are generally less mutated than non-modified proteins (FC = 0.85, *p* <1 × 10^–5^), suggesting that modified proteins are more sensitive to mutations. However, PTM proteins also show higher expression in the TCGA pan-cancer dataset (mean expression FC = 4.00, *p* <10^–300^, Wilcoxon test). Hence their lower mutation frequency may be also explained by transcription-coupled DNA repair [12]. In summary, mutations in lysine PTM sites are positively selected in cancer genomes and therefore are functionally important.

Second, we examined the functional impact of PTM-related mutations relative to protein domains and disordered sequence. SNVs in acetylation and ubiquitination sites are significantly concentrated in protein domains, suggesting that many PTM-associated substitutions directly impact protein structure and activity (FC = 1.15, *p* <1 × 10^–5^, bootstrap test). Analysis of disordered sequence predictions from DISOPRED2 software [39] shows that ubiquitination sites are enriched in structured regions (FC = 1.17, *p* <10^–300^, Fisher’s test), while acetylation sites are evenly distributed in structured and disordered sequence (Fig. 1b). In contrast, phosphorylation sites are primarily found in disordered regions [18]. Thus PTMs differ in their preferences toward protein structure and their mutational impact needs to be analyzed separately due to differences in background mutation distributions. The most frequently PTM-mutated protein domains include histones, the DNA-binding domain of TP53, and domains of unknown function associated with regulatory genes such as the DUF902 domain in the histone acetyltransferase EP300 [55]. Thus lysine PTM mutations may modulate protein function through regulatory switches in structured domains.

Third, we studied evolutionary sequence conservation of PTM-associated mutations relative to other missense mutations using phastCons 46-way gene conservation scores. SNVs found within lysine PTM sites (including ±7 flanking residues) target residues are more strongly conserved than those of non-PTM SNVs (FC = 1.08, *p* <1 × 10^–5^, bootstrap test, Fig. 1c). This relationship holds when separating SNVs into disordered and structured sequences (FC = 1.04, *p* <1 × 10^–5^; and FC = 1.09, *p* <1 × 10^–5^, respectively). We also scored PTM-related mutations by integrating results from five predictors of variant function (SIFT [56], PolyPhen2 [57], LRT [58], PhyloP [59], MutationTaster [60]), as collected by the Annovar software [36]. We scored each SNV on a 0 to 5 scale by counting the methods that consider that variant deleterious according to thresholds curated by the dbNSFP database [61]. SNVs found within PTM sites are predicted to be deleterious more often than other missense SNVs (Fig. 1d). This difference is most evident in high-impact mutations with consensus predictions from all five tools (odds ratio = 1.52, *p =* 4.6 × 10^–17^, Fisher’s exact test).

Together, the three observations suggest positive selection and functional importance of PTM-related mutations in cancer genomes. As ubiquitination and acetylation regulate core processes such as protein degradation and chromatin state, mutations in PTM sites may rewire signaling networks, leading to altered phenotypes important in cancer development.

### ActiveDriver highlights known and candidate cancer genes with PTM-specific mutations

To better understand the distribution and function of PTM mutations in cancer genes, we utilized the ActiveDriver mutational significance model [17]. ActiveDriver identifies genes enriched in PTM-specific mutations using a Poisson regression model that estimates mutational significance from protein disorder, direct and flanking PTM residues, and site density.

**Fig. 2:**
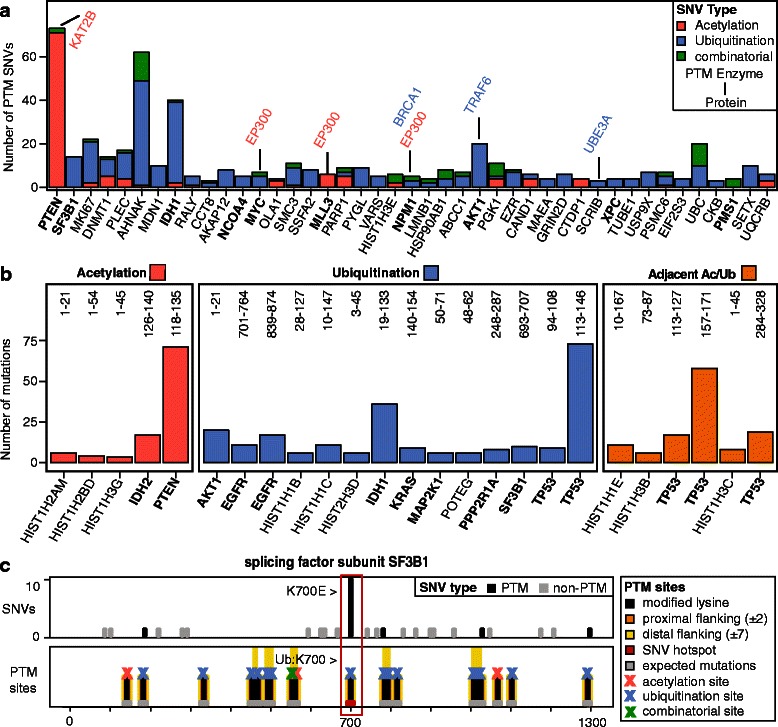
Cancer genes with significant mutations in PTM sites. **a** ActiveDriver predicts cancer driver genes with significant co-occurrence of PTM sites and mutations. Genes are ranked by statistical significance. Known PTM enzymes associated with mutated PTM sites are shown on top of *bars*. Known cancer genes are highlighted in *boldface*. Combinatorial mutations shown in *green* affect lysines that are both acetylated and ubiquitinated. **b** PTM-associated sequence regions with recurrent cancer mutations (more than five SNVs). Sequence coordinates are shown on top of *bars*. Known cancer genes are highlighted in *boldface*. Mutations shown in *orange* are adjacent to both acetylation and ubiquitination sites. **c** Cancer mutations in splicing factor subunit SF3B1 (*top panel*) significantly associate with PTM sites (*bottom panel*). The ubiquitination site K700 is disrupted by the recurrent cancer mutation K700E

Composite analysis of 12 cancer types revealed 43 genes with significant enrichment of PTM-specific SNVs (*p* <0.05, Fig. 2a, Additional file 4). Ten genes are confirmed cancer drivers according to the Cancer Gene Census database [62] (*p* = 2.0 × 10^–7^, custom permutation test), suggesting that mutations in these cancer genes involve alterations of PTM networks. For example, the metabolic enzyme IDH1 carries 36 mutations in residue R132 of the catalytic domain causing altered enzymatic activity and histone demethylation [62, 63]. Our analysis associates these mutations to PTM sites (*p* = 4.47 × 10^–3^ from ActiveDriver) as IDH1 is ubiquitinated at K126. This oncogene may be regulated by a combinatorial PTM mechanism as the hotspot also overlaps with other PTMs, including an adjacent phosphorylation site at Y139 [18]. The gene with the most significant *p* value in the list is the phosphatase PTEN, where a mutation hotspot at R130 disrupts the active pocket of the phosphatase and the arginine required for catalytic activity [64]. ActiveDriver highlights this mutation due to acetylation sites K125 and K128 targeted by the acetyltransferase PCAF (KAT2B); acetylation at these sites are associated with decreased PTEN activity [65]. The ActiveDriver gene list also includes histones as well as PTM-related enzymes like kinases and acetyltransferases. Pathway analysis with g:Profiler [46] shows significant enrichment of several processes, including DNA binding (*p* = 5.0 × 10^–6^), cell cycle regulation (*p* = 5.9 × 10^–3^), and metabolism (*p* = 6.3 × 10^–3^).

To investigate if our analyses could be due simply to mutation clustering, we analyzed clustering of mutations across all genes using OncoDriveClust [41], and found that three of 43 genes from ActiveDriver analysis exhibited statistically significant mutation clustering (IDH1 *p* = 1.2 × 10^–8^, PTEN *p* = 6.0 × 10^–4^, and AKT1 *p* = 5.1 × 10^–9^). Thus, most of the genes ActiveDriver finds are not explained by mutation clustering. We also asked how many of our results depend on our flanking region definition and repeated ActiveDriver analysis by only focusing on central modified lysines. We found six significant genes (SF3B1, AHNAK, TP53, MAP2K1, PYGL, ATPIF1; FDR *p* <0.05), five of which are found in our analyses of ActiveDriver and mutation recurrence.

Several PTM substrates highlighted by our analysis are associated with well-studied enzymes, such as EP300, TRAF, and PCAF, for which pharmacological inhibitors are increasingly available. Enrichment of known cancer genes and pathways validates our results. Follow-up experiments will be required to confirm these findings, as ActiveDriver is primarily a hypothesis generation tool that integrates functional information with mutations in known and candidate cancer genes. Site-specific interactions with PTM enzymes are still relatively unmapped, but better characterization of these networks may lead to discovery of new druggable targets with cancer specific mutations.

### Most frequent PTM-associated mutations involve TP53, the AKT1 kinase, and the histone HIST1H3B

Next, we analyzed PTM sites with the most frequent mutations in the pan-cancer dataset. We retrieved continuous PTM-related sequence regions and annotated these to cancer types, PTM classes, and upstream enzymes. This approach is complementary to ActiveDriver as it also highlights proteins that are likely not found by ActiveDriver due to lack of PTM-specific mutational enrichment. These may include short proteins with relatively large PTM regions and frequently mutated genes with alterations within and outside PTM regions. However, these genes are likely important in cancer due to frequent mutations.

We found 25 distinct PTM-associated mutation hotspots with more than five SNVs, including frequently mutated cancer driver genes such as *TP53* (AA 113-146, n = 73) and *IDH1* (AA 119-133, n = 65) (Fig. 2b). Some PTM sites are also substrates of cancer-related epigenetic enzymes such as *EP300* and *KAT2B*. These genes include several cases that provide proof-of-principle of our hypothesis of PTM mutations in cancer. One well-studied example occurs in TP53 where 17 cancer samples of primarily breast and colorectal cancer have SNVs affecting residue K120. This residue is a combinatorial PTM site with evidence of acetylation and ubiquitination, and a substrate site of the acetyltransferase *KAT8*, important during DNA damage response. Disruption of this PTM through *KAT8* knockout or K120 mutagenesis leads to loss of TP53 mediated apoptosis [66]. Another well-studied cancer mutation we identify is the substitution E17K in *AKT1* (PKB) kinase of the PI3K pathway, observed across 20 samples including 18 breast tumors. This mutation creates a ubiquitination site targeted by the ubiquitin ligase TRAF upstream of the ubiquitination site K14. This site has been found to be readily ubiquitinated, promoting membrane localization and over-activation of the oncoprotein AKT1 [67, 68]. Lastly, our analysis highlights the histone site H3.3-K27, which was recently identified to undergo K27M mutation in rare and rapid onset pediatric brain cancers [69]. In normally functioning cells, methylation at H3K27 has been associated with reduced gene expression. Acetylation at H3K27 is believed to be antagonistic to H3K27 methylation and has been associated with increased gene expression. K27M substitutions at this site cause global reduction in the repressive histone mark of trimethylation (H3K27me3), increasing gene expression and promoting tumorigenesis [70]. We observe mutations in eight samples targeting the K27 site across the *HIST1H3* gene family, primarily within uterine carcinoma samples. Thus, several recurrent cancer mutations we identify are already confirmed in the literature to be functionally related to lysine PTMs, supporting our list of candidate genes.

### Mutations in splicing factor subunit SF3B1 associate with disrupted ubiquitination

*SF3B1* is the second most significant gene with PTM-specific cancer mutations (FDR *p* = 3.2 × 10^–6^ from ActiveDriver, Fig. 2c). While the function of *SF3B1* in cancer is not well established, its mutations are frequently observed in myelodysplastic syndromes [71]. The gene is involved in pre-mRNA processing and splicing, as it encodes subunits of the U2 small nuclear ribonucleoprotein (snRNP) complex and the minor spliceosome complex. Our analysis highlights a recurrent K700E substitution found in nine breast cancer samples that replaces a central ubiquitinated lysine and thus disrupts modification of the protein at that site. Individual mutations are also found near other ubiquitination sites of *SF3B1* (K182, K333, K785), suggesting that altered ubiquitination of the protein is important in cancer.

While little is known about the specific role of ubiquitination of *SF3B1* at K700, this PTM type is involved in spliceosome assembly and function. Ubiquitination mediates protein-protein interactions of snRNP complexes [72], and replacement of wild-type ubiquitin with a non-functional mutant ubiquitin disrupts spliceosome assembly and leads to decreased mRNA splicing [73]. Multiple alignment analysis of homologous protein domains shows that the K700E substitution is less deleterious than randomly generated missense mutations [71]. Most cancer variation in *SF3B1* in the TCGA dataset involves missense mutations, while stop codon and frame-shift mutations are not seen. The two observations suggest that the ubiquitination-associated mutation K700E in *SF3B1* may change protein function while retaining protein structure. Further, Maguire et al. recently showed that K700E mutations likely affect splicing in breast tumors [74]. We propose that loss of ubiquitination at K700 in *SF3B1* leads to altered spliceosome assembly and causes aberrant splicing in cancer.

### Pathway analysis of PTM mutations highlights specific metabolic and signaling pathways, chromatin remodeling, and the APC/C complex

Cancer is driven by alterations of hallmark biological pathways that provide cells with selective advantages during tumor growth [1]. Thus different mutations within the same pathway can lead to similar functional outcomes. To discover pathways with frequent PTM-related mutations and to better interpret rare mutations, we performed a pathway enrichment analysis. We searched for cellular processes and protein complexes, represented as gene sets, that possess unexpectedly high rates of PTM-specific mutations compared to background missense mutation rates. We studied biological processes from Gene Ontology [43], pathways from Reactome [44], and protein complexes from CORUM [45].

**Fig. 3:**
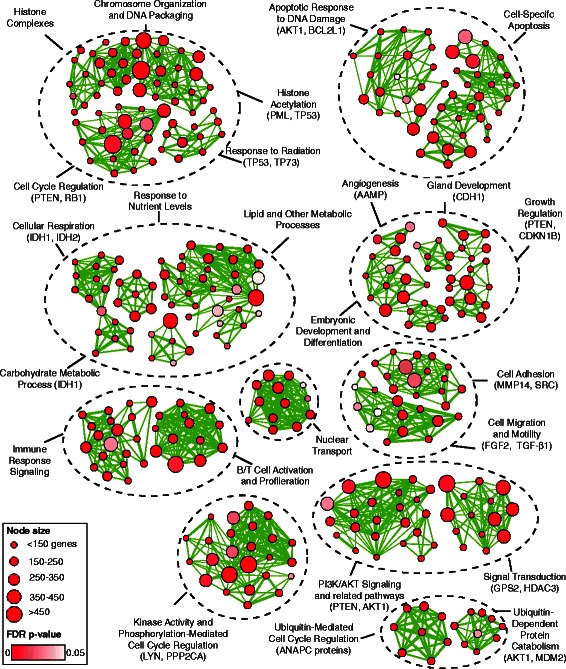
Processes and pathways with frequent cancer mutations in PTM sites. *Enrichment Map* shows a network of processes and pathways with over-representation of mutations in acetylation and ubiquitination sites. *Nodes* represent processes and pathways, and *edges* connect those with many common genes. Similar processes are grouped into functional themes and the most frequently mutated genes are *highlighted*

In total, we identified 587 pathways with significant enrichment of mutations in acetylation and ubiquitination sites (FDR *p* <0.05, Poisson test). We visualized these results as an Enrichment Map [47] and highlighted major functional themes with the most frequently mutated genes (Fig. 3). The top 50 statistically significant gene sets cover cancer hallmark processes including cell adhesion, *PI3K*-*AKT* signaling, apoptosis, cell cycle regulation, and response to DNA damage. Chromatin remodeling processes and histone complexes are also found. Besides recurrently mutated proteins *PTEN*, *AKT1*, and *TP53*, representing nearly half of the enriched GO terms, others with PTM-specific mutations are apparent, such as MDM2, the ubiquitin ligase involved in *TP53* regulation, *CDKN1B*, a cyclin-dependent kinase inhibitor, *AAMP*, an angiogenesis-associated migratory protein, and multiple proteins involved in signal transduction (*GPS2*, *HDAC3*, *LYN*, *PPP2CA*). The multi-subunit anaphase-promoting complex (APC/C) involves 35 SNVs related to acetylation and ubiquitination sites (*p* = 1.19 × 10^–3^). APC/C functions as a ubiquitin ligase that blocks mitosis by marking cell cycle proteins for degradation and its deregulation is linked to genomic instability in cancer cells [75]. As the APC/C complex is also regulated by PTMs, we propose that infrequent cancer mutations in PTM sites drive tumors by deregulating the structure and function of the complex and impacting cell proliferation pathways.

In summary, pathway analysis of mutations in PTM sites identifies recurrent and rare mutations that may affect protein switches in pathways. Deeper investigation of these findings will help elucidate novel mechanisms of cancer biology and pharmacologically relevant mutations.

### Network analysis reveals survival-associated protein modules and suggests that PTM-related mutations relate to poor prognosis

Next, we aimed to characterize PTM-associated mutations in the context of cancer patient survival. Protein-focused survival analysis of specific mutations is challenging, as only few proteins have sufficient mutation frequency to compare patients with PTM-associated mutations within cancer types. Thus, we performed a network module survival analysis using the HyperModules algorithm [52] to cluster infrequently mutated proteins into connected network modules with higher mutation frequency and significant survival associations. The HyperModules algorithm incorporates a permutation-based control procedure that performs clustering with survival data on networks with randomly shuffled mutations to estimate significance of modules detected in true data. For this analysis, we constructed a PTM-specific protein interaction network using the BioGRID database [49], comprising physical interactions between modified proteins and PTM enzymes such as acetylases and ubiquitin ligases (Additional file 5).

**Fig. 4:**
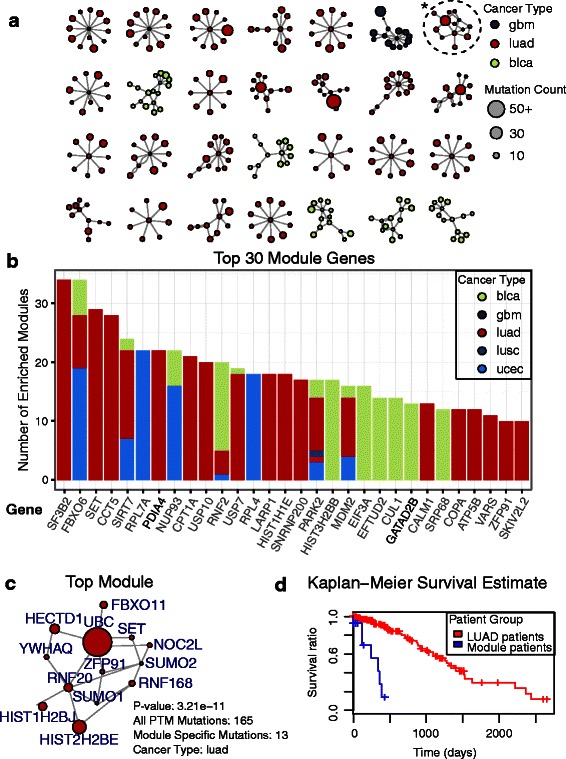
PTM-associated network modules with patient survival correlations. **a** Analysis of a large protein-protein interaction network with HyperModules reveals protein modules where PTM-specific mutations correlate with reduced patient survival. The top 28 modules with lung and brain cancer mutations are shown. Node size corresponds to the number of PTM mutations per gene. **b** Survival-linked interaction modules often comprise overlapping sets of genes. The top 30 genes from modules are shown with colors representing modules discovered for different cancer types. **c** Top module of interest comprises 13 PTM-associated mutations in lung cancer. The module is indicated with an *asterisk* on panel (**a**). **d** Lung cancer patients with mutations in the module of interest have significantly lower survival compared to other lung cancer patients

We carried out HyperModules analyses for 12 cancer types and identified 132 network modules of proteins where PTM-specific mutations indicate significant differences in patient survival (Fig. 4a, FDR *p* <0.05, permutation tests, Additional file 6). Interestingly, all but one module indicate reduced patient survival, suggesting that certain PTM-associated mutations occur in more aggressive tumors. To examine the individual proteins within these partially overlapping and cancer type-specific modules, we ranked proteins by their frequency of occurrence in discovered modules (Fig. 4b). The top protein SF3B2, with five individual mutations primarily occurring in ubiquitination sites of lung adenocarcinoma patients, is a component of the U2 spliceosome pathway, suggesting it plays a similar role to the SF3B1 protein described above. Also included are two histone genes mutated in lung and bladder carcinomas; the HIST1H1E histone expressed across all somatic tissues, and the replication-dependent histone HIST3H2BB, as well as several proteins related to gene expression (*p* = 3.4 × 10^–2^), RNA binding (p = 2.18 × 10^–3^), and ER protein processing (5 × 10^–2^). Our network-guided integration of mutations with patient survival information helps highlight genes and pathways for study as candidate biomarkers. This strategy is useful to interpret sets of rare mutations that converge on common systems and pathways.

### Mutations in the RNF20-HISTH2BE-HISTH2BJ interaction module of ubiquitin signaling associated with transcriptional regulation of proto-oncogenes

One of the top modules from the HyperModules analysis contains several genes associated with transcriptional regulation of oncogenes. The module comprises 13 genes (FBXO11, HECTD1, HIST1H2BJ, HIST2H2BE, NOC2L, RNF168, RNF20, SET, SUMO1, SUMO2, UBC, YWHAQ, ZFP91) with 13 PTM-associated mutations distributed across 13 lung adenocarcinoma (LUAD) patients or one gene mutation per patient (Fig. 4c). Survival analysis shows that mutations in the module associate with markedly lower survival of patients compared to other LUAD patients (log-rank *p* = 3.21 × 10^–11^, permutation test *p* = 0.039, Fig. 4d). The central protein of the module RNF20 is the major E3 ligase that performs ubiquitination of H2B histone proteins. While ubiquitination of H2B histones are associated with the transcriptional regulation of many genes, only a subset of these are regulated by RNF20-mediated histone ubiquitination [76]. Among these genes are several oncogenes including MYC and FOS. Furthermore, RNF20 depletion is associated with transcriptional regulation of cell migration and tumorigenesis [76]. We hypothesize that mutations affecting this module disrupt the ubiquitination of H2B substrate level leading to upregulation of oncogenes and tumorigenesis.

## Discussion and conclusions

Traditional methods of cancer driver discovery primarily focus on mutational frequency to identify sites of positive selection. Our approach extends this strategy by considering prior knowledge about cellular mechanisms. We assume that positive selection in cellular networks indicates their importance in cancer biology. This approach gains statistical power by collecting mutations across multiple positions or genes into a network for input into a single statistical test. By identifying mutations in protein sites that mediate molecular interactions and determine protein activation, inhibition, or degradation, we uncover specific network-related mechanisms that potentially drive cancer. We also reveal rarely, but site-specifically, mutated candidate genes hidden among abundant passenger mutations. Our results represent a resource of mechanistically detailed hypotheses that can be experimentally tested to validate cancer driver genes. Further, the identification of PTM-related enzymes involved in a cancer driver process helps identify approved and experimental drugs targeting these enzymes that may be useful cancer treatments, as we previously showed for the PRC2 complex that methylates histones in ependymoma [77].

Several considerations limit the interpretation of our findings. First, as PTMs occur tissue-specifically and are mostly discovered through high-throughput technologies, our site collection is incomplete and is expected to contain false positives. Second, some sites and mutations may be inactive in cancer cells as our analysis only considers the longest isoform of all proteins. Third, our network analysis utilizes the entire collection of known interactions of PTM enzymes and proteins with PTM sites. We do not consider cellular context such as enzyme activation or co-localization with substrate proteins, as this information is generally not available within cancer samples. We instead rely on identifying signs of positive selection to highlight important PTMs, but this still may fail for PTMs affected by a low number of mutations. Fourth, acetylation and ubiquitination site information is still emerging. As more data become available, in particular proteome-wide measurements in cancer genomics efforts, a number of these limitations will be addressed and we are likely to discover more cancer mutations with PTM-associated function. Fifth, as different types of PTM sites are often clustered in protein sequence, some mutations affecting lysine PTMs may in fact alter phosphorylation or other modifications. Lastly, a number of mutant genes identified by ActiveDriver, such as PTEN, have not been characterized in the context of PTM-mutations and require experimental follow-up for validation.

Positive selection of PTM sites in cancer genomes indicates their involvement in cancer-relevant processes. In the human population, PTM sites undergo specific negative selection and are enriched in diverse mutations of inherited disease [24]. These observations emphasize the importance of PTM sites and associated networks in physiology, development, and disease. Current approaches for interpreting missense SNVs largely focus on sequence conservation, population frequency of variation, and AA properties of substituted residues [5, 78]. Integration of PTM data with the above information provides additional functional evidence not available in traditional methods.

### Ethics approval and consent to participate

Not applicable.

### Consent for publication

Not applicable.

## Additional files

Additional file 1: DISOPRED2 predicted disordered regions in FASTA format (1 = disordered, 0 = ordered). (FASTA 10762 kb) Additional file 2: CCDS longest isoform sequences in FASTA format. (FASTA 10762 kb) Additional file 3: Table of cancer mutations in PTM sites. (CSV 1742 kb) Additional file 4: Table of genes with significant PTM-specific mutations from ActiveDriver. (CSV 2 kb) Additional file 5: Table of network of PTM proteins and interacting PTM enzymes used in HyperModules analysis. Interactions of acetylation proteins and ubiquitination proteins are shown separately. (CSV 377 kb) Additional file 6: Table of protein interaction modules with PTM-specific mutations and survival correlations from HyperModules. (CSV 42 kb)

## Abbreviations

AA: Amino acid(s)
APC/C: Anaphase-promoting complex
CCDS: Consensus Coding Sequence
CPLA: Compendium of Protein Lysine Acetylation
FC: Fold change
FDR: False discovery rate
HATs: Histone acetyltransferases
HDACs: Histone deacetylases
ICGC: International Cancer Genome Consortium
K: Lysine
LUAD: Lung adenocarcinoma
PTMs: Post-translational modifications
snRNP: Small nuclear ribonucleoprotein
SNVs: Single nucleotide variants
TCGA: The Cancer Genome Atlas
